# A Study of the Veterinary Surgeons’ Register of Pennsylvania

**Published:** 1898-01

**Authors:** John J. Repp

**Affiliations:** Veterinary Department, University of Pennsylvania, Philadelphia


### A STUDY OF THE VETERINARY SURGEONS' REGISTER OF PENNSYLVANIA.

BY JOHN J. REPP,
VETERINARY DEPARTMENT, UNIVERSITY OF PENNSYLVANIA, PHILADELPHIA.

UNDER the direction of Dr. Leonard Pearson, State Veterinarian of Pennsylvania, I have at intervals during the last few months been engaged in the compilation of a record of the veterinarians of Pennsylvania. This has given me an opportunity to acquire considerable familiarity with the status of the profession in this Commonwealth. There appear on the Veterinary Surgeons' Registers of the sixty-seven counties of the State 1770 names. In only one county—Pike—have no registrations been made. The highest number—154—is in Philadelphia County.

Of the total number of names registered 120 are duplicates, leaving 1650 distinct registrations. Of these we may safely deduct 250

as numbering those who have died, removed from the State, relinquished practice, or registered as castrators only. This leaves 1400 men who are to a greater or less extent in the practice of veterinary medicine and surgery. Of this number, 400, or 28.5 per cent. of the total number probably in practice, are graduates of seventeen different recognized schools. It is probable that not over 375 of the 400 graduates are now in active practice.

The first law regulating practice of veterinary medicine in Pennsylvania became operative April 11, 1889. This law required all those who desired from that time forward to practise veterinary medicine or castration to make registration in the office of the prothonotary of some county in the State. The requisite qualifications on the part of the applicant for such registration were that he had practised in the State since April 11, 1884, or had graduated from a recognized veterinary school, and that he pay a fee of one dollar to the prothonotary. The law did not demand that the one registering as veterinary surgeon be a graduate. The period for registry was open for a limited time only—*i.e.*, to January 1, 1890. Practically all of the non-graduates, and all of the graduates in practice at that time, registered under this act. The Legislature of 1891 again opened the registry on the same terms as before, extending the period to January 1, 1892. This led to a considerable increase in the number of non-graduates in practice.

Some have argued that the enactment providing for security in practice to non-graduates was a mistake, but on trial it has proven itself a wise procedure. In the first place it is unlikely that the passage of a more stringent law could have been accomplished, for it would have been unjust to stock-owners in those communities where no graduate was located and where the non-graduate was the only available aid the stockmen could secure. Furthermore, a law seeking to expel from an established practice a man who depended upon it for a livelihood would most likely have been declared unconstitutional, for it lacks conformity with the "Bill of Rights."

The non-graduate practitioner has been a gradually decreasing necessity. We foresee the day when he will be no longer needed, yet it is undeniable that he has made the development of veterinary medicine possible, and for this he deserves much credit. Looking back to the early epochs of our country's history, we can discern the method by which the practice of veterinary medicine was evolved in Pennsylvania.

Before the days of veterinary schools, when A. found one of his animals overtaken by the misfortune of accident or disease, and felt himself unable to treat it, he requested B. to render such service as was within his power. If B. manifested certain skill and met with success in treating this case, he would be called upon by C., D., E., etc., when they found themselves in similar unfortunate circumstances. In this way B. acquired a local reputation and some skill as a veterinarian. The same process was repeated in other parts of the State. In exceptional cases these men improved every opportunity within reach, studied their cases and the available literature assiduously, and thus became quite proficient. Eventually a graduate here and there appeared upon the scene. As these qualified men proved their superiority, and their worth became recognized, they gradually displaced the existing practitioner in the treatment of sick animals. It has been a continuous contest between the graduate and the non-graduate with the former ever and increasingly in the ascendency.

The legislative enactment of 1895, requiring that one desiring to enter practice in this State shall be a graduate, shall have had a three-years' course at a recognized veterinary school, and shall successfully undergo examination by the State Board of Veterinary Medical Examiners, effectually puts up the bars against the admission to the profession of any unqualified man. This leaves the State with about 400 graduate veterinarians, which number is gradually increasing, and about 1000 non-graduates, which number is rapidly decreasing. It is safe to predict that within twenty-five years the non-graduate veterinarians will have so dwindled away as to be virtually a nonentity, and the conditions will have so matured that these men will have reached the end of their usefulness. Also the number of graduates will have so increased as to be able to attend to all the demands of the people upon the profession. Until that time we will not be ready to dispense entirely with non-graduate service. The gradual process of evolution now properly established will finally terminate in a beautiful adjustment in the Keystone State.

There are numerous influences which continually operate to expand the field for the competent veterinarian. As the non-graduates of this State, about 1000 in number, retire from practice, at least 500 vacancies for graduates will be made. Allowing twenty-five years for their retirement would create places each year for twenty graduates.

It must also be noted that it is not alone the elimination from practice of the existing practitioners that increases the demand for skilful veterinarians; but the ever-increasing live-stock industry, the awakening of the people to the expediency of better service, the requirements of the various States and the nation for skilled veterinary service, the positions constantly being created in institutions of learning for teachers of veterinary science, are all factors tending to render the prospect bright. Certainly schools of veterinary medicine, and young men undecided as to what profession to enter, ought to find much encouragement in these observations.

Practice of veterinary medicine in Pennsylvania is on as good basis as that of any other State, while it far surpasses the majority. Those States which have inefficient laws regulating the practice of veterinary medicine, or which lack such law, should make its enactment a matter of the gravest consideration. So long as men are allowed to practice at will, because there is no law to specify who shall and who shall not engage in practice, no advance will be made toward the standard which is requisite in order to place veterinary medicine on a parity with the other professions. There is a tendency for unqualified men to remove themselves from the States where they are under the ban of the law to those States where they are not so restricted. Finally, the States which are the last to make such legislation will be surfeited by incompetent men presuming to be capable of coping with the various animal ailments.

It will be argued that the non-graduate is fully able to deal successfully with all problems of pathology and therapeutics which commonly present themselves. However, this is a mistake. The man who has had a thorough training in a good school is the only one whom it is wise to employ. The value of our live-stock is too great to entrust its care to any but the most skilful. The truth that the best is the cheapest must gain recognition from all who will give the subject sufficient thought. This is an age of improvement, and we must look for it here as in other departments of our national economy. Such legislation not only is of value to the veterinarian, but great benefit will also accrue to the people at large. It is demanded by broad considerations of public policy.

It is estimated that preventable diseases of animals cause losses in Pennsylvania of $6,000,000 to $10,000,000 annually. This great drain on the wealth of the State can be repressed only by placing the care of the health of animals entirely in the hands of well-equipped men.

Not of any less importance is the fact that the veterinarian must assume an ever-increasing responsibilty in regard to the health of our human population. Many diseases are communicable from the animal to the human species, and the only way in which these conditions can be met is to properly deal with such diseases in the lower animals. Further, the complexity and technicality of the problems which these ailments present for solution make it imperative that in order to deal with them a man must have the most liberal scientific education.

Public health organizations everywhere are realizing that collaboration between the practitioner of human medicine and the veterinarian is the most direct way to the determination of methods for prevention of many diseases; consequently, we find members of these two professions working side by side, each recognizing the essentiality of the other.

Every State owes its people some legislation leading to the gradual elimination of incompetent veterinarians and the placing of veterinary medicine and sanitation in the care of qualified graduate veterinarians.

---

SURGEON-Major Semple, attached to the Army Medical School at Netley, England, recently scratched his hand with a hypodermic needle when immunizing a horse for the preparation of serum against Malta fever. Shortly after this he developed the symptoms of the fever, and his blood when examined gave a typical reaction of the micrococcus of Malta fever when diluted 1–1000. As an act of poetic justice, the horse which he was immunizing has been bled, and has supplied two injections of its serum into the immunizer. The patient is responding to the treatment and gives promise of speedy recover.—*Medical News.*

A Bandera County, Texas, stock-raiser is the owner of a mare mule with a sucking colt.

The well-known Chestnut Hill Stock-farm of Mr. Mitchell Harrison, near Philadelphia, met with the great loss by fire of all its barns and sheddings for horses, early in November. Fortunately all the live-stock were saved, including the great hackney stallion Wildfire, with many of his get. Mr. Harrison will at once rebuild, and will construct the most complete sanitary buildings possible, in order that the best system of light, ventilation, and drainage may be secured. Mr. Harrison has retained the services of Prof. Leonard Pearson, whose experience and advice will be utilized in preparing the plans and completing the construction of the building.

